# Exploring Movement Impairments in Patients With Parkinson's Disease Using the Microsoft Kinect Sensor: A Feasibility Study

**DOI:** 10.3389/fneur.2020.610614

**Published:** 2021-01-06

**Authors:** Ditte Rudå, Gudmundur Einarsson, Anne Sofie Schott Andersen, Jannik Boll Matthiassen, Christoph U. Correll, Kristian Winge, Line K. H. Clemmensen, Rasmus R. Paulsen, Anne Katrine Pagsberg, Anders Fink-Jensen

**Affiliations:** ^1^Child and Adolescent Mental Health Center, Mental Health Services - Capital Region of Denmark & Faculty of Health Science University of Copenhagen, Copenhagen, Denmark; ^2^Section for Image Analysis and Computer Graphics, DTU Compute, Technical University of Denmark, Copenhagen, Denmark; ^3^Hofstra Northwell School of Medicine, Hempstead, NY, United States; ^4^The Zucker Hillside Hospital, New York, NY, United States; ^5^Department of Child and Adolescent Psychiatry, Charité Universitätsmedizin, Berlin, Germany; ^6^Department of Neurology, Bispebjerg University Hospital, Copenhagen, Denmark; ^7^Psychiatric Centre Copenhagen (Rigshospitalet), Copenhagen, Denmark; ^8^Laboratory of Neuropsychiatry, University Hospital Copenhagen, Copenhagen, Denmark

**Keywords:** Parkinson' s disease, hypokinesia, movement disorder, technology, computer assisted diagnosis

## Abstract

**Background:** Current assessments of motor symptoms in Parkinson's disease are often limited to clinical rating scales.

**Objectives:** To develop a computer application using the Microsoft Kinect sensor to assess performance-related bradykinesia.

**Methods:** The developed application (*Motorgame*) was tested in patients with Parkinson's disease and healthy controls. Participants were assessed with the Movement Disorder Society Unified Parkinson's disease Rating Scale (MDS-UPDRS) and standardized clinical side effect rating scales, i.e., UKU Side Effect Rating Scale and Simpson-Angus Scale. Additionally, tests of information processing (Symbol Coding Task) and motor speed (Token Motor Task), together with a questionnaire, were applied.

**Results:** Thirty patients with Parkinson's disease and 33 healthy controls were assessed. In the patient group, there was a statistically significant (*p* < 0.05) association between prolonged time of motor performance in the *Motorgame* and upper body rigidity and bradykinesia (MDS-UPDRS) with the strongest effects in the right hand (*p* < 0.001). In the entire group, prolonged time of motor performance was significantly associated with higher Simson-Angus scale rigidity score and higher UKU hypokinesia scores (*p* < 0.05). A shortened time of motor performance was significantly associated with higher scores on information processing (*p* < 0.05). Time of motor performance was not significantly associated with Token Motor Task, duration of illness, or hours of daily physical activity. The *Motorgame* was well-accepted.

**Conclusions:** In the present feasibility study the *Motorgame* was able to detect common motor symptoms in Parkinson's disease in a statistically significant and clinically meaningful way, making it applicable for further testing in larger samples.

## Introduction

Parkinson's disease (PD) is a progressive, degenerative movement disorder (1). The neuropathology of PD is characterized by loss of dopamine neurons in the substantia nigra resulting in dysfunction of the nigrostriatal pathway, which lead to perturbations of control and regulation of intentional motor movement. Bradykinesia, rigidity and resting tremor are the cardinal motor symptoms of PD (2). Parkinsonian bradykinesia is the very core symptom and correlates with loss of dopaminergic deficiency (3, 4); it involves difficulties in planning, initiating and executing movements and difficulties in performing various tasks (5). As the disease progresses, postural instability often develops as a fourth cardinal symptom (6). Standard quantitative assessments for evaluating PD bradykinesia include the modified bradykinesia rating scale (7) as well as the Unified Parkinson's Disease Rating Scale (UPDRS), which assesses both motor and non-motor symptoms of PD (8).

When blocking the nigrostriatal pathway by D2-receptor antagonists, i.e., with antipsychotics, symptoms similar to the ones observed in idiopathic PD can occur. Antipsychotic-induced parkinsonism is characterized by bradykinesia, rigidity and (variable) tremor, which reverse upon antipsychotic discontinuation (9, 10). In clinical practice, antipsychotic-induced motor side effects are usually assessed by clinical evaluation. However, a number of rating scales for the evaluation of motor side effects exist, including the Abnormal Involuntary Movement Scale (AIMS) (11), Simpson-Angus Scale (SAS) (12) and the Barnes Akathisia Rating Scale (BARS) (13). Another commonly used rating scale is the UKU Side Effect Rating Scale (UKU is an acronym for the Danish name “Udvalg for Kliniske Undersøgelser,” Task Force for Clinical Investigations) (14). Although all the mentioned rating scales have undergone thorough scientific validation, the fact that the rating scales are observer-based inherently requires adequate training of clinicians in their use and makes these scales vulnerable to inter-observer variability (15–17). Hence, objective methods to detect and quantify movement disorders are needed. Besides overcoming the issue of inter-observer variability, objective technology-based tools may well be usable for home monitoring of symptoms.

Computerized analysis of human movements has been investigated for more than three decades (18). Until recently, human motion capture (the process of registering motion) has required an extensive setup, typically involving several cameras, structured light projectors, and special markers attached to the different relevant body parts that are tracked. With the introduction of the Microsoft Kinect system in 2010, a low-cost motion tracking technology has become available.

We have developed a simple game-like application using the Microsoft Kinect, in which the user is asked to push buttons on a computer screen in a specific sequence, while the application tracks the movement of the major joints in the upper body. The objectives for this work was to test the feasibility of the *Motorgame* in bradykinetic persons in both clinical and non-clinical environments, and to study the degree to which the *Motorgame* can complement the traditional observer-based rating scales of PD related bradykinesia.

We hypothesized that the Kinect would be acceptable to patients and that higher scores on rigidity and bradykinesia related tests would generally be associated with prolonged time of motor performance (TOMP).

More specifically, we hypothesized that:

1. Prolonged TOMP in the *Motorgame* will be associated with higher
  1. MDS-UPDRS scores
  2. SAS rigidity scores
  3. UKU bradykinesia scores
2. Shortened TOMP in the *Motorgame* will be associated with higher
  1. Token Motor Test motor speed scores
  2. Symbol Coding Task information processing speed scores.

## Materials and Methods

### Participants and In- and Exclusion Criteria

This study included patients (age > 18 years) with idiopathic PD (ICD-10 G20.9) (2, 19) and a maximum score of 2.5 on the Hoehn & Yahr scale (i.e., posturally stable) (20). Exclusion criteria were dementia, current psychosis, or on current antipsychotic treatment. The patient group was sought to be matched 1:1 to healthy controls on age and sex. Exclusion criteria in the healthy control were: Parkinson's disease, dementia, current psychosis, or lifetime antipsychotic treatment.

### Recruitment

Patients were recruited in the Capital Region of Denmark at the Department of Neurology, Bispebjerg University Hospital (*n* = 3), and from neurologists in primary sector (*n* = 27).

Healthy controls (*n* = 33) were recruited through local contacts, tennis clubs, and senior centers in the Capitol Region. The technical work was initiated in January 2013, and the first demonstration model was ready for data collection in June 2013. Data collection was initiated in February 2014 and proceeded until August 2016.

### Data and Acquisition

The data for this study comes from the Microsoft Kinect v1 sensor (21), which we refer to as the Kinect or Kinect sensor. The Kinect contains a RGB (Red Green Blue device-dependent color model) camera, an infrared camera and an infrared projector. The infrared camera and projector make it possible to estimate the depth of each pixel acquired by the RGB camera. Thus, the video stream that comes from the Kinect at 30 frames per seconds includes the standard video from the RGB camera, and for each pixel we get a *D*-value, which is an estimate of the distance from the Kinect to the point seen by the camera. This type of data is referred to as RGB-D video. One of the main innovations of the Kinect is the skeletal tracking algorithm (22). The skeletal tracking algorithm is based on the Random Forest prediction algorithm (23). When using the Kinect sensor, the data provided by the algorithm consist of a multivariate time-series of measurements, where positional measurements for hands, wrists, elbows, shoulders, neck and head are provided as 3D world coordinates. We only used coordinates from the upper body in this study.

We implemented a game-like environment in order to record series of movements of the participants. We refer to this environment as the *Motorgame*.

### Description of the Motorgame

**Figure 1:**
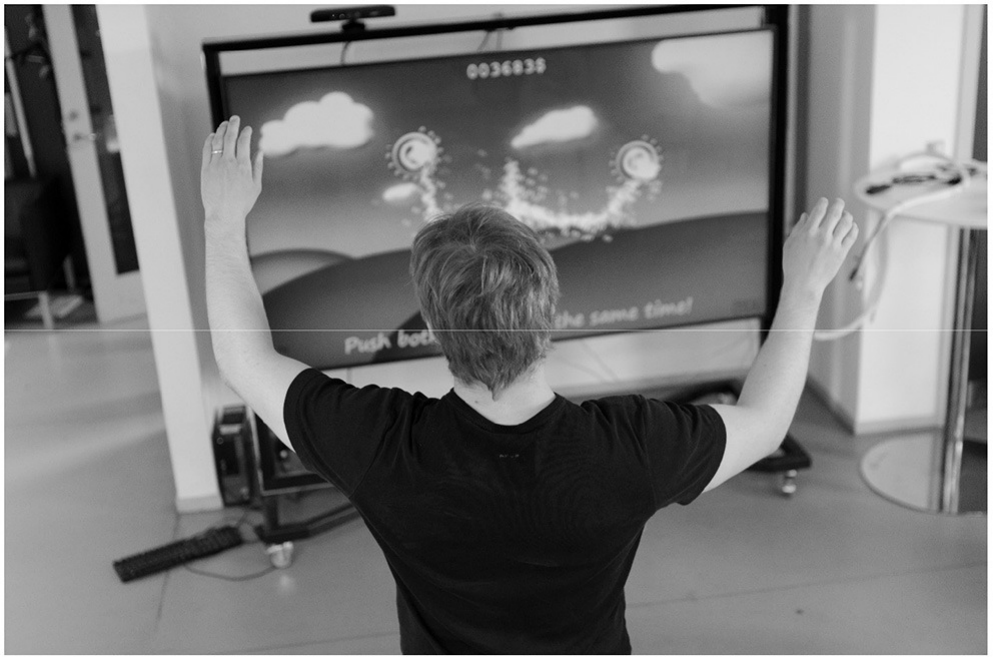
A participant playing the Motorgame. A Kinect sensor is placed on the top of the television/computer and tracks the participant's movements. The participant's pose is mirrored as a stickman figure on the screen.

**Figure 2:**
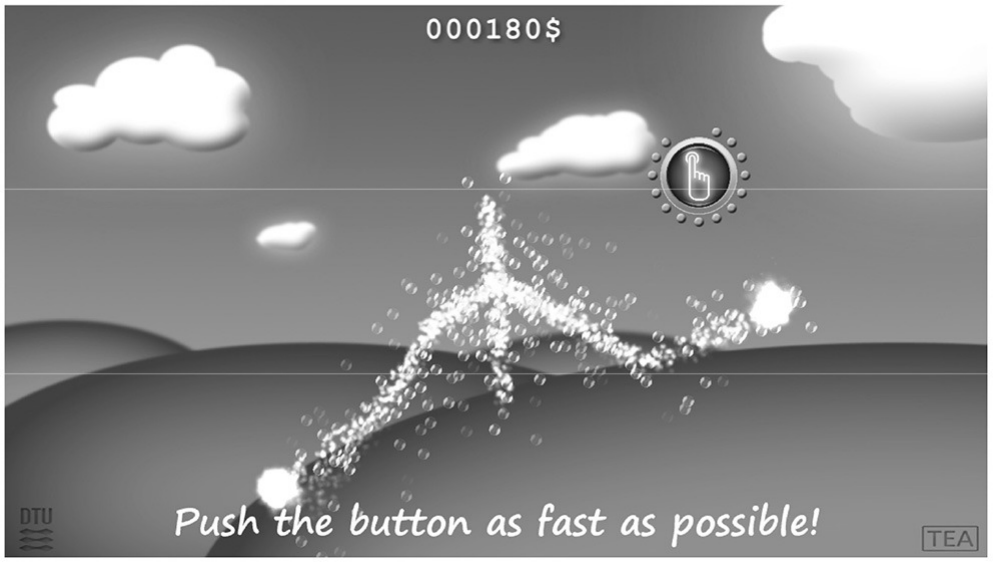
Level 1 in the Motorgame from the participant's perspective. The participant is moving its right hand upwards to reach the blue button visible on screen.

**Figure 3:**
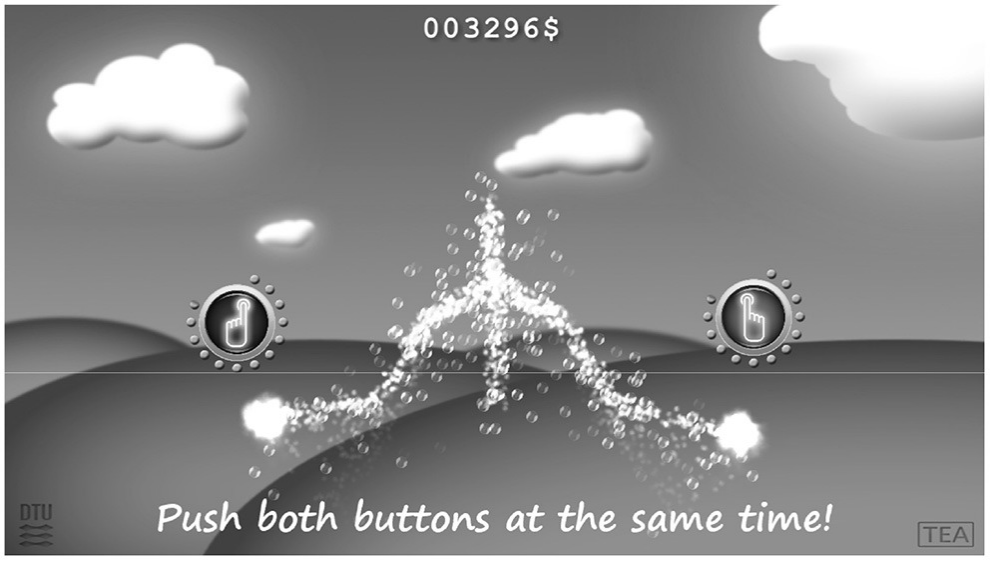
Level 2 in the Motorgame from the participant perspective. Now the participant has to touch two buttons simultaneously.

The *Motorgame* was developed, so that the participant observed the upper body of a stickman figure on a computer screen that mirrored the movements of the participant (See Figure 1). First, the participant was asked to place themselves at a distance between 2 and 3 m from the screen for optimal recording conditions. The participant was then asked to stretch out their arms. This was done for calibrating the arm length of the stickman figure. After this procedure, a message appeared on the screen stating that the participant should try to finish the upcoming tasks as fast and precisely as possible. Then the following tasks were split up into three levels. Before each level a welcome screen appeared, indicating that the participant had to perform a different task at the next level (See Figure 2, and Figure 3). The participant needed to perform similar movements of the hands repeatedly, but the design of random appearance of the button made it hard to *learn* this task. A score was displayed on the top of the screen where the participant was awarded a higher score if they finished the task fast. In order to avoid interruptions during the recording, a training session was performed before the actual recording session.

### Description of Data

When a participant played the entire *Motorgame*, the data were recorded in a comma separated text file. Each entry in the file corresponds to one frame from the RGB-D video, where the frames were recorded 30 times per second. The RGB-D video data were stored in a separate file. The *screen coordinates* were the 2D coordinates of the joints as seen on the screen when playing the game.

### Assessments

All participants were assessed with standard neurological examination. All patients with PD were assessed in “on” state with a mean time period from last administration of usual medication of 161 min (range 0–540 min). Only patients with PD were assessed with the Movement Disorder Society Unified Parkinson's Disease Rating Scale (MDS-UPDRS) (8). All participants were assessed with the UKU Side Effect Rating Scale (14); assessing symptoms similar to antipsychotic-induced side effects, including hypokinesia) and the Simpson-Angus Scale (12) (SAS; assessing symptoms similar to antipsychotic-induced parkinsonism). In addition, two subtests from the Brief Assessment of Cognition in Schizophrenia (BACS) (24) assessing attention and information processing speed (Symbol Coding Task) and motor speed (Token Motor Task) were conducted. All participants were assessed with the *Motorgame*. Finally, participants filled out a questionnaire developed by the authors for the present study, a five-point Likert scale about comprehensibility of the instructions, personal evaluation, and preferred choice of test, which assessed their opinion about the *Motorgame* compared to the additional clinical assessments.

All assessments of the patients were administered by one medical doctor (DRU), who prior to this study received training in the UPDRS at the Department of Neurology, Bispebjerg University Hospital as well as training in the clinical rating scales for the evaluation of motor side effects by a post-doctoral-level psychiatrist (JN) at the Department of Psychiatry, Aalborg University Hospital. Assessment of the healthy controls were administered by DR and one medical student (SKR). DRU and SKR had attended a systematic BACS training program by two post-doctoral-level neuropsychologists (BF and JRMJ), in which the training procedure (25) was delineated in agreement with Dr. Richard Keefe from NeuroCog Trials.

### Data From the Motorgame

Data from the *Motorgame* were multivariate time-series of varying length, hence a single play of the *Motorgame* generated a lot of data. For the reason of testing the value of the measurement with respect to bradykinesia, the only variable we extracted for this analysis was the time it took to finish each of the tasks in level 1 of the *Motorgame*. For a given participant we obtained 22 variables. These measurements can be seen as 22 repeated measurements for a given participant, but due to the differences in the tasks, i.e., how far the participant had to move their hand, these measurements were inherently different. Hence, no corrections for multiple comparisons were made, but the difference between measurements was accounted for in the statistical model by assigning a fixed effect to each task.

As the data did not fit a Gaussian distribution, natural logarithm transformation was used. Due to the log transformation, parameters can approximately be seen as percentwise increase/decrease in time it took to finish the tasks. For example, a parameter estimate of 0.06 for male participants indicated that it took 6% longer time to finish the tasks compared to females. This approximation is good for low values of parameters due to the Taylor-expansion of the natural logarithm having the coefficient value of one for the linear term.

### Statistical Methods

To analyze the data we used a linear mixed effect model (26) using the package lmer (27) for the R-programming language (28) that provided *p*-values for the fixed effect in the model. The model also included a general mean term and the error was assumed to be independent and identically distributed from a normal distribution.

$$\begin{array}{l} ln\left(y\right)=\mu +T_{j+}Cx_{ijC}+Sx_{ijS}+H_{ijH}+Ax_{ijA}+Wx_{ijW}+SYx_{ijSY}\\ \;+{\text{ε}}_{i+}\text{ε} \end{array}$$

The terms in the model were, *y* the response (time in seconds it took to finish a single task in level 1), and on the right hand-side we had in the following order: μ as a general mean, T_j_ mean for each of the 22 tasks in level 1, *C* parameter for the clinical score, where *x*_*ij*_ was the value for that measurements on participant *i* in task *j*. *S* was the sex, *H* was the height, *A* was the age, *W* the weight and *SY* the result of the Symbol Coding Task. The last two terms were ε_*i*_, the random effect for participants, and finally the general error term.

All tests were two-sided with alpha = 0.05 and without correction for multiple comparisons, as this was a feasibility study with exploratory analyses of the correlation between the clinical and Motorgame scores for the assessment of PD related bradykinesia.

## Results

Demographic and disease specific characteristics are shown in Table 1. Thirty patients with PD and 33 healthy controls were assessed. All patients and healthy controls completed the entire session of the *Motorgame*. As fewer healthy control males consented to participate in the study, the intended matching for sex was not achieved and the male proportion was significantly higher in the patient group than in the healthy control group (60.0% vs. 30.3%, *p* = 0.018). All 30 patients (100%) were right-handed. All, but one, (97%) in the healthy control group were right-handed. Results of the clinical assessments showed significant differences between the two study groups (Table 2). A significantly higher proportion of the healthy controls managed to complete the Token Motor Task without modifications (pushing or tipping the tokens) than in the PD group (*p* = 0.001). Likewise, the PD group had significantly lower mean scores on the Token Motor Task (indicating reduced motor speed in the fingers) vs. healthy controls (25.5 ± 18.1 vs. 39.9 ± 13.4, *p* = 0.003). Furthermore, patients had significantly higher mean SAS total scores (indicator of rigidity; *p* < 0.001) and hypokinesia UKU scores (indicator of bradykinesia; *p* < 0.001) compared to the group of healthy controls. No significant difference in mean scores (SD) in the Symbol Coding Task (information processing speed) was found between the PD group (37.53 (13.62) and the healthy control group (41.42 (10.15); *p* = 0.201).

**Table 1:** Demographic and disease specific characteristics.

	**Patients with Parkinson's Disease (*n* = 30)**	**Healthy controls (*n* = 33)**	***P*-value**
Males, *n* (%)	18 (60.0)	10 (30.3)	0.018^a^
Age in years, mean (SD)	70.1 (6.7)	69.7 (6.1)	0.787^b^
Family history of Parkinson's Disease, *n* (%)	8 (26.7)	2 (6.1)	0.025^a^
Hoehn and Yahr score, median (IQR)	2 (2–2)	–	–
Mean duration of Parkinson's Disease in months, mean (SD)	45.7 (34.0)	–	–
L-dopa equivalent dose Tomlinson CL 2010 mg, mean (SD)	868.4 (1902.2)	–	–
Physical activity hours/week, mean (SD)	6.6 (5.5)	7.7 (4.6)	0.388^b^
Computer games hours/week, mean (SD)	0.17 (0.91)	0 (0.0)	0.306^b^

a chi^2^ test;

b unpaired t-test; “-” not applicable; SD Standard Deviation; IQR Interquartile Range.

**Table 2:** Clinical assessments.

	**Patients with Parkinson's Disease (*n* = 30)**	**Healthy controls (*n* = 33)**	***P*-value**
BACS Token Motor Task (completers), *n* (%)	17 (56.67)	31 (93.94)	0.001^a^
BACS Token Motor Task score, mean (SD)	25.53 (18.13)	39.89 (13.44)	0.003^b^
BACS Symbol Coding Task, mean (SD)	37.53 (13.62)	41.42 (10.15)	0.201^b^
SAS total score, mean (SD)	0.87 (0.70)	0.13 (0.19)	<0.001^b^
Hypokinesia (item 2.3 UKU), mean (SD)	1.17 (0.65)	0.09 (0.29)	<0.001^b^

a chi^2^ test;

b ANOVA test; SD Standard Deviation.

### Mixed Model Analysis

The results from the mixed model analyses of the effect of motor MDS-UPDRS items scores (part III) on the time of motor performance in the *Motorgame* are seen in Table 3. Since MDS-UPDRS was only assessed in patients with PD, all controls were assigned the value zero for the measured variables in this analysis, which is consistent with prior studies using the MDS-UPDRS in healthy controls (29, 30). Compared to zero, all motor items in the MDS-UPDRS corresponding to bradykinesia and rigidity in the upper body, except for finger tapping on the left and hand movements on the left, had a significant (*p* < 0.05) effect on prolonging the time of motor performance in the *Motorgame*. The strongest effects were from *finger tapping on the right, hand movements on the right*, and *rotation of the right hand* (items of bradykinesia; *p* < 0.001). A negative moderating effect of Symbol Coding Task scores was found for all motor MDS-UPDRS items (*p* < 0.001), i.e., a higher (better) information processing score corresponded to a shortened time of motor performance. No moderating effects of age or body weight were found. Significant (*p* < 0.05) moderating effects of male sex (negative, i.e., shorter performance time) and height (positive, i.e., longer performance time) were found in the MDS-UPDRS items of finger tapping on the right, hand movements on the right, and rotation of the right hand, but not regarding the remaining motor MDS-UPDRS items scores.

**Table 3:** The effect of motor items in the MDS-UPDS on the time of motor performance in the Motorgame.

	**MDS-UPDRS item**	**Sex**	**Age**	**Height**	**Weight**	**Symbol Coding Task**
3.3a Rigidity Neck	0.0401* (0.0191)	−0.105 (0.0524)	−0.000552 (0.00307)	0.00517 (0.00349)	−0.00132 (0.00174)	–0.00555** (0.00161)
3.3b Rigidity Right Arm	0.0447* (0.0185)	−0.0994 (0.051)	0.000343 (0.00306)	0.00542 (0.00343)	−0.00136 (0.00171)	–0.00509** (0.00162)
3.3c Rigidity Left Arm	0.0372 (0.019)	−0.0916 (0.0516)	0.000325 (0.00313)	0.00525 (0.00351)	−0.00133 (0.00175)	–0.0051** (0.00167)
3.3d Rigidity Right Leg	0.0351* (0.0156)	−0.0946 (0.0511)	−0.000163 (0.00306)	0.00543 (0.00346)	−0.00174 (0.00171)	–0.00508** (0.00164)
3.3e Rigidity Left Leg	0.0364* (0.0165)	−0.0876 (0.0509)	−0.000624 (0.00306)	0.00515 (0.00348)	−0.00159 (0.00171)	–0.00497** (0.00166)
3.4a Finger Tapping Right Hand	0.0528** (0.0149)	–0.135** (0.0502)	0.000949 (0.00291)	0.00799* (0.00328)	−0.00203 (0.0016)	–0.00517** (0.0015)
3.4b Finger Tapping Left Hand	0.0308 (0.0161)	−0.103 (0.0527)	−0.000768 (0.00309)	0.00581 (0.00349)	−0.00114 (0.00177)	–0.00562** (0.00161)
3.5a Hand Movements Right	0.0583** (0.0162)	–0.116* (0.0486)	0.00106 (0.0029)	0.00757* (0.00325)	−0.00163 (0.0016)	–0.00508** (0.0015)
3.5b Hand Movements Left	0.024 (0.0167)	−0.0872 (0.0523)	−0.000534 (0.00314)	0.00546 (0.00357)	−0.00123 (0.00181)	–0.00567** (0.00165)
3.6a Pronation-Supination Movements of Hands Right	0.06** (0.0163)	–0.12* (0.0486)	0.00066 (0.00287)	0.00804* (0.00326)	−0.002 (0.00159)	–0.0053** (0.00149)
3.6b Pronation-Supination Movements of Hands Left	0.0302* (0.0148)	−0.0841 (0.0511)	−0.000763 (0.00308)	0.00535 (0.00349)	−0.00129 (0.00174)	–0.00564** (0.0016)
3.12 Postural Stability	0.0201 (0.0229)	−0.0839 (0.053)	−0.000243 (0.00321)	0.00642 (0.00359)	−0.00183 (0.00178)	–0.00601** (0.00164)
3.13 Posture	0.0438 (0.0218)	−0.0969 (0.0519)	−0.00101 (0.00308)	0.00592 (0.00348)	−0.00145 (0.00174)	–0.00573** (0.0016)
3.14 Global Spontaneity of Movement (Body Bradykinesia)	0.0419* (0.0183)	–0.111* (0.0525)	−0.000369 (0.00305)	0.00515 (0.00347)	−0.000732 (0.00178)	–0.00517** (0.00163)

Results are logtransformed (natural logaritme). Each parameter estimate is presented with the standard deviation in parenthesis (SD).

* p < 0.05;

** p < 0.001.

### Mixed Model Analysis–Clinical Assessments

As seen in Table 4, SAS items scores corresponding to bradykinesia and rigidity, as well as the hypokinesia UKU score, had a significant (*p* < 0.05) positive effect on the time of motor performance in the *Motorgame*. Furthermore, a negative, moderating effect of Symbol Coding Task scores was found in all SAS items (*p* < 0.001). No significant effects of Token Motor Task (motor speed), duration of PD and hours of weekly physical activity were found. No moderating effects of age, height or weight were found.

**Table 4:** The effect of clinical assessment scores (Simpson Angus Scale, UKU and Token Motor task) and disease related characteristics on the time of motor performance in the Motorgame.

	**Clinical assessment score**	**Sex**	**Age**	**Height**	**Weight**	**Symbol Coding Task l**
SAS-1 Gait	0.0613^*^ (0.025)	–0.1028^*^ (0.051)	−0.00004 (0.003)	0.0050 (0.003)	−0.0008 (0.002)	–0.0046^*^ (0.002)
SAS-2 Arm drop	0.0717^*^ (0.024)	−0.0938 (0.049)	−0.0009 (0.003)	0.0040 (0.003)	−0.0009 (0.002)	–0.0046^*^ (0.002)
SAS-3 Shoulder shaking	0.0766^*^ (0.023)	–0.1016^*^ (0.049)	−0.0001 (0.003)	0.0041 (0.0033)	−0.0008 (0.002)	–0.0044^*^(0.002)
SAS-4 Elbow rigidity	0.0441^*^(0.017)	–0.1044^*^ (0.051)	0.0002 (0.003)	0.0053 (0.003)	−0.0012 (0.002)	–0.0052^*^ (0.002)
SAS-5 Wrist rigidity	0.0742^*^ (0.026)	–0.1020^*^ (0.050)	−0.0004 (0.003)	0.0052 (0.003)	−0.0014 (0.002)	–0.0053^*^ (0.002)
SAS-6 Leg pendulousness	0.0369^*^ (0.013)	−0.0955 (0.050)	−0.0006 (0.003)	0.0051 (0.003)	−0.0013 (0.002)	–0.0053^*^ (0.002)
SAS-7 Head dropping	0.0431^*^ (0.019)	–0.1071^*^ (0.052)	−0.0007 (0.003)	0.0051 (0.003)	−0.0012 (0.002)	–0.0056^**^ (0.002)
SAS-8 Glabella tap	0.0323^*^ (0.011)	−0.0813 (0.051)	0.0005 (0.003)	0.0046 (0.004)	−0.0010 (0.002)	–0.0049^*^ (0.002)
SAS-9 Tremor	0.0162 (0.018)	−0.0824 (0.053)	−0.0010 (0.003)	0.0059 (0.004)	−0.0019 (0.002)	–0.0061^**^ (0.002)
SAS-10 Salivation	0.0987 (0.056)	−0.0929 (0.052)	−0.0002 (0.003)	0.0061 (0.004)	−0.0014 (0.002)	–0.0056^*^ (0.002)
2.3 Hypokinesia (UKU)	0.0665^*^ (0.022)	–0.1128^*^ (0.050)	−0.0008 (0.003)	0.0047 (0.003)	−0.0002 (0.002)	–0.0052^*^ (0.002)
Token Motor Task	−0.0012 (0.001)	−0.0852 (0.054)	−0.0005 (0.003)	0.0061 (0.004)	−0.0017 (0.002)	–0.0054*(0.002)
Duration of Parkinson	0.0009 (0.000)	−0.1010 (0.053)	−0.0000 (0.003)	0.0072 (0.003)	−0.0018 (0.002)	–0.0056*(0.002)
Physical activity	0.0001 (0.003)	−0.0791 (0.054)	−0.0006 (0.003)	0.0062 (0.004)	−0.0019 (0.002)	–0.0061^**^ (0.002)

Duration of Parkinson's Disease (PD) is in months. Physical activity is average number of hours per week.

* p < 0.05;

** p < 0.001.

### Acceptance of the Motorgame

The application was well-accepted and preferred over the clinical rating scales and the BACS subtests by 76% in the healthy control group and 53% in the patient group.

## Discussion

Initially developed as an entertainment device, e.g., used for dancing games, the Kinect sensor is now in widespread research use, including neuro-rehabilitation (31), assessment of post-stroke movement impairment (32), and classification of movements during active video gaming (33).

In our study of 30 patients with PD and 33 healthy controls, we found a highly significant association between prolonged time of motor performance in the *Motorgame* and higher scores of MDS-UPDRS items related to right hand movements (bradykinesia). In contrast, this association was not found in items related to left hand bradykinesia. Asymmetrical onset of PD symptoms has been shown to be more likely to occur in the dominant hand (34). Patients with dominant-side onset do more often report initial bradykinesia compared to patients with non-dominant-side onset (35). Since all, but one, participants in our study were right-handed this might be the explanation to the side difference found in our results.

Bradykinesia has been shown to correlate strongly with a broad cluster of PD motor symptoms (36). Furthermore, 18 F-DOPA PET brain scans in PD patients with predominantly hypokinesia and rigidity motor symptoms correlate significantly with dopaminergic depletion in the striatum (37). In our study, we found a statistically significant association between prolonged motor performance and upper-body rigidity (MDS-UPDRS).

The same associations between time of motor performance and bradykinesia and rigidity were found in relation to the clinical rating scales: i.e., prolonged time of motor performance in the *Motorgame* was related to higher rigidity SAS scores and UKU hypokinesia. However, surprisingly, we did not find a significant association between time of motor performance in the *Motorgame* (assessing gross motor skills) and motor speed (Token Motor Task; assessing fine motor skills). A possible explanation might be that the *Motorgame* is a more complex motor test demanding a high level of eye-hand coordination and cognitive skills. This was confirmed by the finding of a significant moderating effect of the Symbol Coding Task on the time of performance of the *Motorgame* (i.e., shortened time of performance was associated with higher/better scores of information processing).

Furthermore, we did not find an association between time of motor performance in the *Motorgame* and the duration of PD. An explanation could be that the included group of patients in this study were all well-medicated, which was reflected by their median (IQR) Hoehn and Yahr score of 2 (2-2) and that the mean (±SD) duration of illness was 45.7 ± 34.0 months.

We found that males had a significantly faster performance time in the majority of the estimates, which is consistent with findings in healthy individuals (38). Whether this difference is further accentuated by PD cannot be analyzed in our study due to the small sample size.

In line with a study of adaptive training/rehabilitation in patients with PD (39), we found a high level of participant acceptance of the *Motorgame*, showing that the *Motorgame* was the preferred choice of test by the majority of the healthy control group (76%) as well as in the patient group (53%).

Overall, our results are consistent with previous studies of the Kinect sensor used as a supplementary assessment for patients with PD. Galna and colleges studied the accuracy of the Kinect in nine PD patients and 10 healthy controls comparing it to the Vicon three-dimensional motion analysis system (40). They demonstrated a high accuracy of the Kinect sensor when measuring time and gross spatial characteristic movements relevant to PD and highly appropriate for distinguishing non-PD subjects from PD patients treated with deep brain stimulation. Likewise, the Kinect sensor has shown high validity regarding gait parameters when validated against a multiple-camera 3D motion capture system (41).

### Strengths and Limitations

The study has several strengths. Firstly, the instrument is low-cost, easily accessible, portable and easy to administer, and does not require expert clinical knowledge to use. Secondly, applicability to the clinical setting was tested and proven on several levels. In terms of practicality, the Kinect-based instrument was easy to set up in hospitals, in private houses, in tennis clubs and senior centers. The test bears potential to be carried out even in intensive care wards and in small examination rooms. Thirdly, the study showed that all PD participants and healthy controls completed the *Motorgame*. Previous studies have shown that videogames for patients with PD should not be made too difficult, in terms of their pace or cognitive complexity (42). Fourthly, by including healthy controls, matched for age, which is one of the most important variables for variation in motor performance (38).

The study has some limitations. Firstly, our *Motorgame* only covers upper extremities and neck and does not distinguish between dominant or non-dominant hand of the participant. Secondly, the version of the Kinect device used in the present study does not have the accuracy to detect tremor, at least not based on the motion trajectories computed by the internal Kinect algorithms. Potentially, tremor related measurements could be extracted from the raw Kinect depth-image information. However, due to the small sample size in this study, experimental data exploration of this kind was not possible. For this reason, we also excluded the tremor related clinical measurements from the analysis. Newer devices with higher accuracy are currently being developed, which potentially might enable tremor detection. Thirdly, healthy controls were not adequately matched to PD patients based on sex, which besides age is a second variable relevant to motor performance (38). However, in our analyses, we covaried for sex, diminishing the effect that age could have had in the findings. Fourthly, healthy controls were not assessed with MDS-UPDRS even though mild parkinsonian signs in this healthy age group would be expected. This has to be taken into account in future studies. Further, we limited ourselves to perform the analyses on a simple meta-variable (the playing time). However, the system is able to gather large amounts of data from the movement patterns of the tested participants. The reason for restricting the analysis to the level 1 task data is the potential problem of overfitting the sparse set of available movement related features such as speed, acceleration and deceleration, and even the use of more automated feature extraction techniques. These aspects should be taken into considerations in future studies.

Fifthly, the sample size in this study is small, thereby enhancing the risk of both type I and type II errors. However, the number of included patients was based on the prestudy power analysis and several of the hypotheses of the study were confirmed.

Sixthly, the PD patients were evaluated in a highly specialized university clinic, while the healthy controls were evaluated in tennis clubs and senior centers. However, 27 (90%) patients were recruited from primary sector and the cohort can be seen as representative for the general population of PD patients. A larger scale study is needed for confirming these findings.

In conclusion, we have presented an easy to use system, the *Motorgame*, with data showing significant associations with currently used clinical motor scores. The concept of using a gamified measurement device showed high acceptability among the participants and high feasibility in both hospital and non-hospital environments. The *Motorgame* offers an accessible objective complementary tool to the traditional observer-based rating scales of motor disturbance symptoms. However, further development is needed to improve the tracking of tremor and motor symptoms in the lower extremities. The present data suggest the potential utility of using portable and accessible systems like this on a much larger scaler and in different patient groups.

## Data Availability Statement

The datasets presented in this article are not readily available due to patient protection privacy. Requests to access the datasets should be directed to the corresponding author.

## Ethics Statement

The study was approved by the Committees on Health Research Ethics for the Capital Region of Denmark and the Danish Health Authority. The Danish Data Protection Agency approved the data collection and data storing. Written informed consent to participate in this study was provided by all participants. Written informed consent was obtained from the individual for the publication of any potentially identifiable images included in this article.

## Author Contributions

DR has initiated the study and collected the clinical data. DR, GE, AA, JM, CC, KW, LC, RP, AP, and AF-J have made important contributions to the study conception, design and protocol. DR, GE, and AA have led the manuscript drafting. DR, AP, AF-J, KW, RP, and GE have been involved in drafting and all authors have critically revised the manuscript. JM, GE, LC, and RP have developed the application for the Kinect sensor. GE has done the mixed model analysis. All authors contributed to the article and approved the submitted version.

## Conflict of Interest

KW has been a consultant and/or advisor to Abbvie and has scientific collaboration with Lundbeck. AF-J has received an unrestricted research grand from Novo Nordisk. CC has been a consultant and/or advisor to or has received honoraria from: Alkermes, Allergan, Angelini, Boehringer-Ingelheim, Gerson Lehrman Group, Indivior, IntraCellular Therapies, Janssen/J&J, LB Pharma, Lundbeck, MedAvante-ProPhase, Medscape, Merck, Neurocrine, Noven, Otsuka, Pfizer, Rovi, Servier, Sunovion, Supernus, Takeda, and Teva. He has provided expert testimony for Bristol-Myers Squibb, Janssen, and Otsuka. He served on a Data Safety Monitoring Board for Boehringer-Ingelheim, Lundbeck, Rovi, Supernus, and Teva. He received royalties from UpToDate and grant support from Janssen and Takeda. He is also a shareholder of LB Pharma. The remaining authors declare that the research was conducted in the absence of any commercial or financial relationships that could be construed as a potential conflict of interest.
